# Bidirectional Mamba with Dual-Branch Feature Extraction for Hyperspectral Image Classification

**DOI:** 10.3390/s24216899

**Published:** 2024-10-28

**Authors:** Ming Sun, Jie Zhang, Xiaoou He, Yihe Zhong

**Affiliations:** 1 College of Computer and Control Engineering, Qiqihar University, Qiqihar 161006, China; 2 Heilongjiang Key Laboratory of Big Data Network Security Detection and Analysis, Qiqihar University, Qiqihar 161006, China

**Keywords:** CNN, Mamba, dual-branch feature extraction, HSI

## Abstract

The hyperspectral image (HSI) classification task is widely used in remote sensing image analysis. The HSI classification methods based on convolutional neural networks (CNNs) have greatly improved the classification performance. However, they cannot well utilize the sequential properties of spectral features and face the challenge of increasing computational cost with the increase in network depth. To address these shortcomings, this paper proposes a novel network with a CNN-Mamba architecture, called DBMamba, which uses a bidirectional Mamba to process spectral feature sequences at a linear computational cost. In the DBMamba, principal component analysis (PCA) is first used to extract the main features of the data. Then, a dual-branch CNN structure, with the fused features from spectral–spatial features by 3D-CNN and spatial features by 2D-CNN, is used to extract shallow spectral–spatial features. Finally, a bidirectional Mamba is used to effectively capture global contextual information in features and significantly enhance the extraction of spectral features. Experimental results on the Indian Pines, Salinas, and Pavia University datasets demonstrate that the classification performance surpasses that of many cutting-edge methods, improving by 1.04%, 0.15%, and 0.09%, respectively, over the competing SSFTT method. The research in this paper enhances the existing knowledge on HSI classification and provides valuable insights for future research in this field.

## 1. Introduction

### 1.1. Background

In the field of remote sensing technology, hyperspectral imaging and synthetic aperture radar [1,2,3] have attracted considerable attention from researchers due to their wide applications in remote sensing imaging. Hyperspectral imaging is the process of acquiring and analyzing the spectral information of objects, ranging from the visible spectrum to the infrared spectrum. Compared to traditional color images, hyperspectral images (HSIs) provide richer details and more spectral information. By capturing the reflection or radiation characteristics of the target object in hundreds of narrow-band spectral channels, hyperspectral imaging can provide detailed information about the composition, structure, and state of the object. It has been widely used in agriculture, environmental monitoring, and ground object classification. In these applications and studies, HSI classification is an important link and has become an active research topic in the fields of remote sensing and Earth observation.

HSI classification aims to classify the pixels in the HSI according to their corresponding land cover categories. However, as the acquisition of HSIs by sensors is often affected by the atmosphere, acquisition angle, and acquisition instruments, it is difficult to accurately identify the land cover categories corresponding to the pixels. Improving the accuracy of classification is therefore a goal that researchers are pursuing.

With the rapid development of deep learning, various HSI classification methods based on deep learning have been proposed to improve the accuracy of classification. Transformer [4], based on the attention mechanism, can respond to valuable regions of the entire image and has become a mainstream backbone network. Taking advantage of the powerful local feature extraction capabilities of convolutional neural networks (CNNs) [5,6,7], many researchers have adopted CNN-Transformer architectures for HSI classification [8,9,10]. Typically, when processing HSI classification tasks, Transformers convert HSI features into longer sequence tasks. However, as indicated by reference [11], Transformers are more likely to capture irrelevant or weakly related information when dealing with sequences, which introduces noise. This further distracts the attention mechanism, preventing it from focusing on the important parts of the input and altering the overall focus. In addition, HSI classification tasks face the problem of same spectrum–different materials [12,13,14], where the same material can exhibit different spectral characteristics. Furthermore, due to the high spatial resolution of HSI, the abundant information in the images increases intra-class variation, such as rooftops with shadows in hyperspectral images. These factors interfere with the attention mechanism when processing hyperspectral image classification, thus affecting classification performance. Motivated by these challenges, we aim to explore alternative strategies to replace Transformers and improve the accuracy of HSI classification.

### 1.2. Related Work

Early work on the HSI classification focuses on traditional classification methods, such as support vector machine (SVM) [15], logistic regression [16], random forest [17], k-means clustering [18], and other methods. For example, Melgani et al. [15] demonstrated that SVM has a high classification potential in the high-dimensional feature space by combining theoretical analysis and experimental research. Bo et al. [19] proposed a classification method based on the joint collaborative representation (JCR) and the SVM model, and adopted a decision fusion technology. The authors used the JCR method to extract mid-features to train the multi-classification SVM model, and improved the classification accuracy through multiplication fusion rules. Zhao et al. [20] proposed a method that combines the spectral gradient, SVM, and spatial random forest (RF) to better describe and capture both the edges and the details of HSIs. Mounika et al. [21] introduced principal component analysis (PCA) into the SVM to reduce the negative impact of the noise band on classification results. The above traditional methods provide multiple effective solutions for HSI classification and promote the development of HSI classification. However, these traditional methods are often prone to more misclassifications, resulting in unsatisfactory classification accuracy.

The rapid development of deep learning technology has had a significant impact on various fields, especially in the field of computer vision, including image classification [22,23,24], object detection [25,26], and semantic segmentation [27]. Popular backbone networks in deep learning include convolutional neural networks (CNNs), recurrent neural networks (RNNs) [28], generative adversarial networks (GANs) [29,30], and graph convolutional networks (GCNs) [31,32]. To extract high-level features of HSI, Chen [33] proposed a new hybrid framework based on PCA, the deep learning architecture, and logistic regression. Since CNNs have a powerful ability to extract high-level semantic features of HSIs, determining how to use CNNs for HSI classification has become an important research topic. Since HSIs contain a large amount of spectral information, Hu et al. [34] used a 1D-CNN to extract spectral features and directly classified HSIs in the spectral dimension. However, this method does not effectively utilize spatial information, resulting in suboptimal classification performance. Makantasis et al. [35] constructed a multi-layer 2D-CNN to reduce the dimension of spectral information and to obtain both the deep and the valuable spatial features. Nevertheless, HSIs contain both 2D spatial information and 1D spectral information, but the methods in [34,35] only handle hyperspectral image (HSI) data from a single feature dimension. This leads to insufficient application of hyperspectral data and failure to fully leverage the powerful feature extraction capabilities of CNNs, which negatively impacts classification results. To make full use of the multi-dimensional characteristics of HSI, He et al. [36] proposed a multi-scale 3D-CNN (M3D-CNN) for HSI classification, which can jointly learn the 2D multi-scale spatial features and 1D spectral features in HSI in an end-to-end manner. In [37], Li et al. proposed a multi-scale deep mid-level feature fusion network (MMFN), which fully integrates the strong complementarity and correlation information between features of different scales and extracts more discriminative features to improve classification accuracy. Although the two methods mentioned above leverage multi-scale features to extract significant information from the feature maps, they also introduce the problem of information redundancy. Hamida et al. [38] employed multiple stacked 3D-CNNs to achieve hyperspectral image classification. While this approach reduces the likelihood of redundant feature extraction, it also increases the computational burden of the model. Furthermore, using only the 2D convolution to extract features will lose the spectral information of the image, and using only the 3D convolution will lose the spatial information of the pixels. If no other strategies or structures are added, a single network model with only the 2D or the 3D convolution will always fail to extract more effective information. Roy et al. [39] proposed a hybrid spectral convolutional neural network (HybridSN), which consists of a spectral–spatial 3D-CNN followed by a spatial 2D-CNN. The 3D-CNN first extracts joint spatial–spectral features, and the 2D-CNN learns more abstract spatial features on top of the 3D-CNN. This method addresses the issue of insufficient data application when using only 2D CNNs, while also utilizing 2D CNNs to replace certain components of 3D CNNs, thereby reducing the number of parameters in the model. However, as the number of the network layers increases, the gradient may become very small and even disappear during the back-propagation process. To solve this problem, He et al. [40] introduced residual networks for image classification. The residual network ensures more comprehensive feature information extraction through skip connections and residual blocks, minimizing the information loss of each convolutional layer. Zhong et al. [41] designed an end-to-end spectral–spatial residual network (SSRN) that uses residual blocks to connect each 3D convolutional layer and uses the features of the next layer to supplement the feature information of the previous layer to improve classification accuracy. Yang et al. [42] proposed a CMR-CNN method for HSI classification that simultaneously utilizes the spatial information and spectral information of pixels, which includes a 3D residual structure for extracting spectral features and a 2D residual structure for extracting spatial features. The above methods all use CNNs and their variants, which show strong capabilities of CNNs in extracting spatial information and local contextual information. However, the above methods still have certain limitations in capturing sequence properties. CNNs are good at extracting local features, but the spectral sequence information in HSIs exhibits long-range dependencies. Relying solely on CNNs may not effectively capture the global spectral–spatial features. In addition, the presence of noise in the spectral sequences can adversely affect classification accuracy, as CNNs may be sensitive to this noise without adequate regularization or preprocessing.

Recently, researchers have introduced Transformer, which is commonly used in natural language processing, to computer vision tasks, showing great potential. Dosovitskiy et al. [43] proposed the Vision Transformer (ViT) model, which uses the self-attention (SA) mechanism to effectively capture the relationship between different positions in the image. By dynamically adjusting the input weights, the model can flexibly capture and utilize the most relevant information and perform well in the field of image processing. Subsequently, more and more Transformer-based methods have been proposed for HSI classification. Liao et al. [44] used Transformer to obtain long-term dependencies between long-distance features and adaptively focus on the characteristics of different regions. They integrated it with CNNs for comprehensive feature extraction and embedded a Gaussian modulation attention module between Conv3D and Conv2D, which can enhance secondary features and suppress the most and least important features. Cao et al. [45] proposed a model (MRViT) that mixed residual convolution with Vision Transformer (ViT). This model used ViT for spectral feature extraction, overcoming the limitation of CNNs that spectral features are difficult to process. Ma et al. [46] proposed a Vision Transformer (VIT) method based on the lightweight Gaussian self-attention (LSGA) mechanism to address the limitations of local modeling and increased computational cost of CNNs in HSI classification. Sun et al. [47] proposed a method called SSFTT, which organically integrates CNN and Transformer, transforms the spectral–spatial features extracted by the convolution layers into semantic tokens, and models the semantic features using Transformer Encoder, making the analysis of land cover characteristics more comprehensive. However, the attention mechanism in the Transformer model has a high computational complexity. As the length of the input sequence increases, the amount of computation increases exponentially, requiring a lot of time and computational memory, and it is easily affected by noise and outliers.

### 1.3. Motivation and Contribution

Motivated by the challenge that Transformer is susceptible to distraction from noise and outliers, we are exploring new strategies to address this issue. Recent research advances have attracted widespread attention on the potential of state space models (SSMs) [48] for modeling long sequences. SSMs can establish long-distance dependencies through state transitions and achieve parallel computing through convolution, so that they can handle sequence problems with near-linear complexity. Mamba [49] is a new class of selective SMM that introduces time-varying parameters into SSM and proposes a hardware-aware algorithm to achieve very efficient training and inference. Inspired by the successful application of Mamba in processing natural language sequences, more and more studies have attempted to apply Mamba to visual tasks. Zhu et al. [50] and Liu et al. [51] successfully applied Mamba to the field of two-dimensional image classification. Similar to transformers, Mamba can capture long-range dependencies but with greater computational efficiency, making it particularly well suited to high-dimensional datasets such as HSI. Currently, many researchers are trying to apply this model to HSI classification. Li et al. [52] proposed a Mamba-based HSI classification model that can simultaneously model long-range interactions across the entire image and adaptively integrate both spatial and spectral information. However, pure Mamba used for HSI classification tasks faces challenges, such as unidirectional modeling and lack of positional awareness. Yang et al. [53] utilized bidirectional reversed CNN pathways to extract spectral features and incorporate a specialized block for spatial analysis. However, this method achieves spatial feature extraction only by stacking 2D CNNs, which may result in insufficient feature extraction and consequently affect the classification performance of the model.

This paper proposes a new network called DBMamba, which includes a CNN-based dual-branch feature extraction module, an HSI feature tokenization module, and a bidirectional Mamba (Bim) encoder module. To make full use of the rich spectral and spatial information of HSI data, a CNN-based dual-branch feature extraction module is used to extract shallow spectral–spatial features. Specifically, the first branch extracts the spectral–spatial features, and the second branch extracts the spatial features. In addition, the features obtained from the two branches are fused in the channel dimension. Next, the fused features are segmented into non-overlapping patches and are flattened. The flattened feature is then fed into the HSI feature tokenization, where it undergoes Gaussian weighting to enhance the focus on key features. In HSI classification, to replace the attention mechanism in Transformers with bidirectional modeling and position awareness, a Bim encoder module is proposed to combine bidirectional SSM and position embedding for global visual modeling and visual position perception of the data, respectively. Specifically, features are extracted from the sequence in both the forward and backward directions, and the output is obtained by adding them after being gated by linear mapping, and finally the classification result is obtained by the multi-layer perceptron head (MLP head).

The main contributions of this paper can be summarized as follows:

- To comprehensively extract spatial and spectral feature information from HSI, we designed a high-performance end-to-end network. This network combines a CNN-based dual-branch feature extraction module, a HSI feature tokenization, and a Bim encoder module. This integration further enhances the classification performance of the CNN-Mamba network.
- In our DBMamba network, we introduce an efficient dual-branch CNN module designed to extract shallow spatial–spectral features. This module arranges a 3D convolutional layer and a 2D convolutional layer in parallel and uses a 2D convolutional layer to fuse the features obtained from the two branches. This module is then combined with a bidirectional Mamba structure to further improve the classification performance.
- Considering the challenge that Transformer is susceptible to noise and outliers in HSI classification tasks, we design the Bim encoder, which effectively improves classification performance by capturing global contextual information from features in both the forward and backward directions. From leveraging CNN networks for shallow feature extraction to using the Bim framework to effectively capture global contextual information in images, the proposed DBMamba enables comprehensive learning of spectral–spatial features in HSIs, significantly improving joint classification accuracy. Experimental verification on three classic public datasets demonstrates the effectiveness of the network framework.

## 2. Materials and Methods

**Figure 1:**
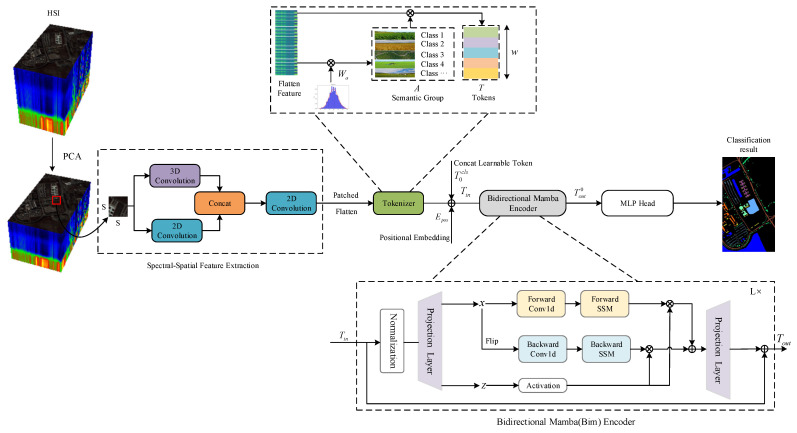
The overall framework of the proposed DBMamba network for the HSI classification.

The section proposes a novel network, i.e., DBMamba, for HSI classification. The overall architecture of the network model is shown in Figure 1. It mainly consists of the following three parts: the CNN-based dual-branch feature extraction module, the HSI feature tokenization module, and the Bim encoder module.

In particular, the CNN-based dual-branch feature extraction module aims to extract shallow spatial and spectral features simultaneously through a dual-branch structure. The HSI feature tokenization module can emphasize the distinction between key features and secondary features, thereby enhancing discriminability. The bidirectional Mamba (Bim) encoder module aims to enhance the ability to model long-range dependencies by processing feature sequences in both forward and backward directions at linear cost.

### 2.1. CNN-Based Dual-Branch Feature Extraction

In the field of HSI classification, CNNs have demonstrated powerful feature extraction capabilities. HSI contains rich spectral and spatial information. It is well known that 3D-CNN and 2D-CNN can not only obtain the joint spectral–spatial features of HSI, but also obtain the spatial dimension information separately. Therefore, the proposed CNN-based dual-branch feature extraction module first uses 3D-CNN and 2D-CNN to extract HSI features.

We represent the original HSI data using a 3D tensor $Z\in {\mathbb{R}}^{m\times n\times k}$, where m×n represents the spatial dimensions of the HSI and k is the number of spectral bands. Each pixel in the HSI belongs to one of the ground object coverage categories. We use $Y=(y_{1},y_{2},\ldots ,y_{L})$ to represent the label of the HSI data, where L is the number of ground object coverage categories in the current area, and each pixel contains k spectral bands. Note that a large number of spectral bands in the HSI data will cause redundancy in the spectral data, affect the accuracy of the HSI classification, and increase the computational complexity. Hence, the PCA operation is first used to reduce the number of spectra bands from k to b, and the HSI data after the PCA are denoted as $Z_{pca}\in {\mathbb{R}}^{m\times n\times b}$, where b is the number of spectra bands after the PCA.

We extract N adjacent 3D blocks $B\in {\mathbb{R}}^{s\times s\times b}$ from the HSI data $Z_{pca}$, and these extracted 3D blocks are used as the input of the entire model, where s×s represents the spatial dimensions of the extracted block, and b is the number of spectral bands of the extracted block. The center pixel position of these extracted 3D blocks is represented by $\left(x_{a},x_{e}\right)$, where 0≤a<m, 0≤e<n. HSI classification classifies each pixel as a certain category in the current area coverage, and the true label of each 3D block is determined by the label of the center pixel. When extracting 3D blocks from edge pixels, padding with (s−1)/2 width is applied to the original HSI data to fully extract pixel information and solve the problem of missing edge pixels. After removing the background information of all 3D blocks, the remaining 3D blocks are divided into a training set and a test set for model training and evaluation.

**Figure 2:**
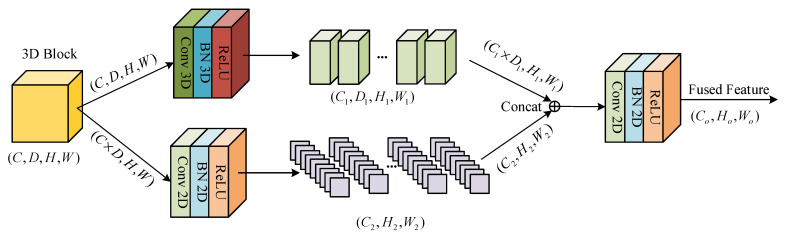
The structure of the CNN-based dual-branch feature extraction module.

After a series of data preprocessing, these 3D blocks are input into the CNN-based dual-branch feature extraction module to extract the shallow spectral–spatial features. As shown in Figure 2, this module contains two sub-modules: 3D and 2D. The 3D module consists of a 3D convolution layer, a BN layer, and a ReLU layer. The convolution kernel is set to 8@ (3 × 3 × 3), and the padding size is set to (0 × 1 × 1). The 3D convolution layer is implemented by applying a 3D convolution kernel to convolve with each individual 3D block. The spectral–spatial features are expressed as follows:

(1) $$v_{i,j}^{x,y,z}=f(\sum_{d}\sum_{\alpha =0}^{H_{i}-1}\sum_{\beta =0}^{W_{i}-1}\sum_{\gamma =0}^{R_{i}-1}\omega_{i,j,d}^{\alpha ,\beta ,\gamma }\cdot v_{(i-1),d}^{\left(x+\alpha ),(y+\beta ),(z+\gamma \right)}+b_{i,j})$$

where f(⋅) is the activation function, $v_{i,j}^{x,y,z}$ represents the neuron at the *j*-th feature map (x,y,z) of the *i*-th layer, and $H_{i}$, $W_{i}$, and $R_{i}$ represent the height, width, and depth of the 3D convolution kernel of the *i*-th layer, respectively, $\omega_{i,j,d}^{\alpha ,\beta ,\gamma }$ is the weight parameter of the *d*-th feature cube at position (α,β,γ), and $b_{i,j}$ is the bias term. In the first branch, 8 3D feature cubes with rich spectral–spatial features are generated after the 3D blocks pass through the 3D module.

At the same time, to make full use of the spatial information, the second branch converts the 3D blocks into 2D data and uses a 2D module to extract spatial features. In the 2D module, it consists of a 2D convolution layer, a BN layer, and a ReLU layer. The convolution kernel is set to 64@ (3 × 3) and the padding size is set to (1 × 1). The spatial features can be expressed as follows:

(2) $$v_{i,j}^{x,y}=f(\sum_{d}\sum_{\alpha =0}^{H_{i}^{\prime }-1}\sum_{\beta =0}^{W_{i}^{\prime }-1}\omega_{i,j,d}^{\alpha ,\beta }\cdot v_{(i-1),d}^{\left(x+\alpha ),(y+\beta \right)}+b_{i,j})$$

where ${H^{\prime }}_{i}$ and ${W^{\prime }}_{i}$ are the height and width of the 2D convolution kernel, $\omega_{i,j,d}^{\alpha ,\beta }$ represents the weight of the *d*-th feature map at position $\left(H_{i}^{\prime },W_{i}^{\prime }\right)$, and $b_{i,j}$ is the bias term. Next, to effectively merge the features obtained from both branches, we first fuse the 3D feature cubes, which carry rich spectral–spatial features, along both the channel and spectral dimensions to obtain 2D data. The resulting 2D data are then concatenated with the 2D spatial feature data obtained from the second branch along the channel dimension. Finally, we use a 2D-CNN to further extract the fused features. The convolution kernel is set to 128@ (3 × 3), and the padding size is set to (1 × 1). The CNN-based dual-branch feature extraction module can be expressed as follows:

(3) $$X_{cancat}=Concat[Conv3D(X_{in},k_{1},p_{1}),Conv2D(X_{in}^{\prime },k_{2},p_{2})]$$

(4) $$X_{out}=Conv2D(X_{cancat},k_{3},p_{3})$$

In Equations (3) and (4), “*Concat*” represents the concatenation operation in the channel dimension, $X_{in}$ represents the 3D blocks, $X_{in}^{\prime }$ represents the 2D data converted from 3D blocks, $X_{cancat}$ represents the fused feature obtained by concatenating the features from the two branches, k represents the size of the convolution kernel, $k_{1}$ is (3 × 3 × 3), $k_{2}$ and $k_{3}$ are (3 × 3), p represents the padding size, $p_{1}$ is (0 × 1 × 1), and $p_{2}$ and $p_{3}$ are (1 × 1).

### 2.2. HSI Feature Tokenization

**Figure 3:**
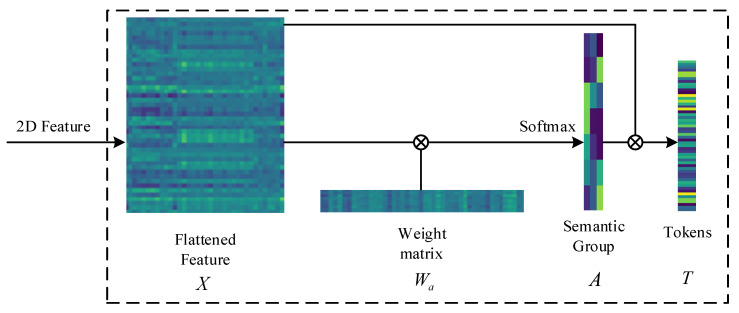
The visualization process of the HSI feature tokenization.

The fused feature extracted by the CNN-based dual-branch feature extraction module carry rich shallow spectral–spatial features. We generate 2D features by patching the fused features. However, the key features and secondary features of the 2D feature have not yet been distinguished and emphasized. The use of Gaussian weighting helps the model prioritize key features by assigning them greater importance based on a Gaussian distribution. This approach ensures that the most critical features receive more attention. Thus, we redefine the 2D features as semantic tokens, which can represent the high-level semantic concepts of the HSI feature. We first flatten the 2D features and represent the flattened feature as $X\in {\mathbb{R}}^{uv\times z}$, where u, v are the height and width of the 2D feature, and z is the number of channels. The final semantic tokens are defined as $T\in {\mathbb{R}}^{j\times z}$, where j is the number of tokens. The tokens can be obtained by the following Equation (5):

(5) $$T=\underset{A}{\underset{︸}{softmax{\left(XW_{a}\right)}^{T}}}X$$

where $W_{a}\in {\mathbb{R}}^{z\times j}$ represents a weight matrix initialized with a Gaussian distribution, and $XW_{a}$ represents element-wise multiplication with dimensions 1 × 1. At this time, the semantic group is represented by $A\in {\mathbb{R}}^{uv\times j}$. Then, we transpose A and apply softmax(⋅) to acquire the relatively important semantics part. The tokens finally are generated by the multiplication of A and X. The operation transforms these features into a new feature space. This transformation is critical for capturing higher-order relationships between features that might not be as apparent in the original space. To provide a clear representation of the tokenization, Figure 3 demonstrates an example of the transformation process layer.

### 2.3. Bim Encoder Module

The semantic tokens generated by the HSI feature tokenization can be represented as $\left[T_{1},T_{2},\cdots ,T_{j}\right]$ and are fed to the Bim encoder module to learn the relationships between high-level semantic features. This module primarily comprises three main parts.

As the first part, to perform the classification task, we add a learnable class token $T_{0}^{cls}$ in the middle of the tokens. Then, the position embedding is used to mark the position information of each semantic. This paper is different from Transformer where the cosine position embedding was used. Instead, we use a learnable position embedding $E_{pos}$ and add it to the semantic tokens, which can better learn the global features of HSI. The operation of adding class token and position embedding can be expressed as Equation (6):

(6) $$T_{in}=[T_{0}^{cls},T_{1},T_{2},\cdots ,T_{j}]+E_{pos}$$

The second and critical part is the Bim encoder. It mainly contains a normalization layer, two projection layers, and SSMs. The SSM is a concept derived from the linear time-invariant system in modern control theory. Many sequence models are generated based on the SSM, such as the structured state-space sequence model (S4) and Mamba. Inspired by continuous systems, a one-dimensional function or sequence x(t)∈ℝ↦y(t)∈ℝ is mapped by a hidden state $h(t)\in {\mathbb{R}}^{N}$, which can be expressed as Equation (7). The system uses $A\in {\mathbb{R}}^{N\times N}$ as the evolution parameter and $B\in {\mathbb{R}}^{N\times 1}$, $C\in {\mathbb{R}}^{1\times N}$ as the projection parameters.

(7) $$\begin{array}{l} h^{\prime }(t)=Ah(t)+Bx(t),\\ y(t)=Ch(t). \end{array}$$

S4 and Mamba are discrete versions of continuous systems, including the time scale parameter Δ, which converts continuous parameters A and B into discrete parameters $\bar{A}$ and $\bar{B}$. The commonly used transformation method is zero-order, which is defined as Equation (8):

(8) $$\begin{array}{l} \bar{A}=\exp(\Delta A),\\ \bar{B}=(\Delta A{)}^{-1}(\exp(\Delta A)-I)\cdot \Delta B. \end{array}$$

After discretization, the discretized version of Equation (7) using the step size Δ can be expressed as Equation (9):

(9) $$\begin{array}{l} h_{t}=\bar{A}h_{t-1}+\bar{B}x_{t},\\ y_{t}=Ch_{t}. \end{array}$$

Finally, the SSM output is calculated through a global convolution, which is expressed as Equation (10):

(10) $$\begin{array}{l} \bar{K}=(C\bar{B},C\bar{AB},\ldots ,C{\bar{A}}^{M-1}\bar{B}),\\ y=x*\bar{K}, \end{array}$$

where *M* is the length of the input sequence *x*, “∗” is the convolution operation, and $\bar{K}\in {\mathbb{R}}^{M}$ is the structured convolution kernel.

The original Mamba was designed for one-dimensional sequence tasks and lacks spatial perception and understanding when processing visual tasks. The Bim encoder uses bidirectional sequence modeling to better handle visual tasks. By merging past and future contextual information together, it can more comprehensively obtain the comprehensiveness of the previous and next sequence information and extract more useful features.

**Figure 4:**
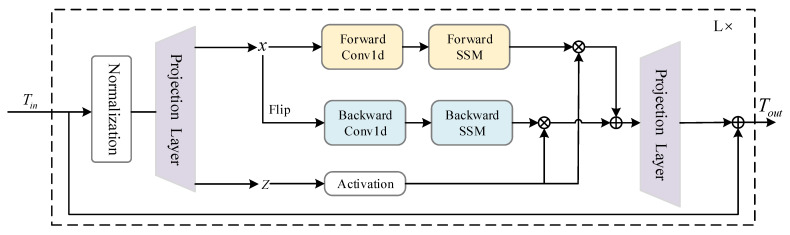
The Bim encoder structure diagram.

As shown in Figure 4, in the Bim encoder, the token sequence with class token and position embedding is first processed by the normalization layer to improve the robustness and stability of the model. Then the normalized data are linearly projected to *x* and *z*, and *x* is processed from the forward and backward directions, respectively. In the forward direction, after the Conv1d operation is performed, $y_{forward}$ is calculated by the SSM. In the backward direction, a flip operation is performed on *x* in the second dimension. Currently, the feature sequence is flipped. The feature originally at the end will be processed first by the SSM to obtain $y_{backward}$. $y_{forward}$ and $y_{backward}$ are gated by *z* and added together to get the output token sequence $T_{out}$. The third part is the MLP head, where the class token $T_{0}^{cls}$ from $T_{out}$ is passed into the MLP head to produce the classification results. The complete procedure of the proposed DBMamba method is shown in Algorithm 1.
**Algorithm 1** Dual-branch feature extraction with bidirectional Mamba model**Input:** Input the HSI data $Z\in {\mathbb{R}}^{m\times n\times k}$ and the ground-truth labels $Y\in {\mathbb{R}}^{m\times n}$; Initialize PCA bands number b = 30, and the size of cube space s = 28; Specify the training sample rate as µ.**Output:** Predicted labels of the test dataset.1: Set batch size to 64, optimizer Adam (learning rate: 5 × 10^−4^), epoch number ε to 100.2: Obtain the data $Z_{pca}\in {\mathbb{R}}^{m\times n\times b}$ after PCA transform.3: Create all sample cubes in the $Z_{pca}$, and divide them into training dataset and test dataset.4: Generate training loader and test loader.5: **for** i = 1 to ε **do**6:  Generate shallow features using the spectral–spatial shallow feature extraction module.7:  Linearly map the extracted 2D shallow spectral–spatial feature maps to obtain a 1D feature vector.8:  Initialize class token and position embedding weights.9:  The middle positions of feature sequences are combined with learnable class tokens and positional embeddings are added to them.10: Perform Bim module.11: Input the middle classification token into the MLP head and identify the labels.12: **end for**
13: Use test dataset with the trained model to get predicted labels.

## 3. Results

### 3.1. Data Description

To verify the effectiveness of the proposed method, experiments were carried out on the Indian Pines dataset, the Salinas dataset, and the Pavia University dataset. Training samples were randomly selected from each dataset. Shares of 10% and 90%, 5% and 95%, 5% and 95% of the three datasets were randomly selected as the training and test sets, respectively. Table 1 lists the land cover categories, the number of training samples, and the number of test samples for the three datasets.

**Figure 5:**
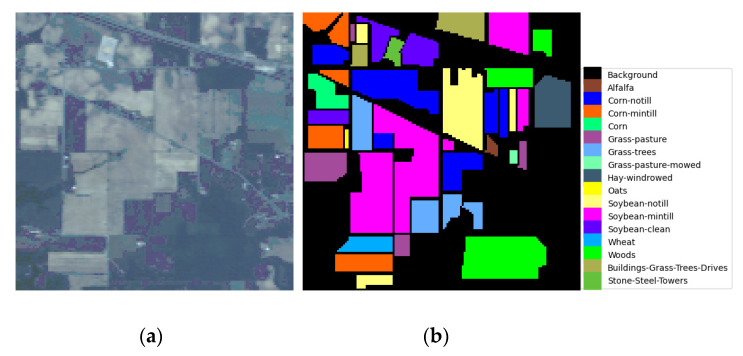
Visualization of the India Pines (IP) dataset. (**a**) Pseudo-color image for the dataset. (**b**) Ground-truth map for the dataset.

*Indian Pines dataset:* The dataset was collected in 1992 by the airborne visible/infrared imaging spectrometer (AVIRIS) sensor over the Indian pine test site in northwestern Indiana and consists of 224 spectral reflectance bands covering the wavelength range of 0.4 to 2.5 microns. The image consists of 145 × 145 pixels and 16 land cover categories. In this experiment, a total of 200 bands were selected, discarding the noise bands [104–108, 150–163], and 220. The pseudo-color image and the ground-truth map are shown in Figure 5a,b, respectively.

**Figure 6:**
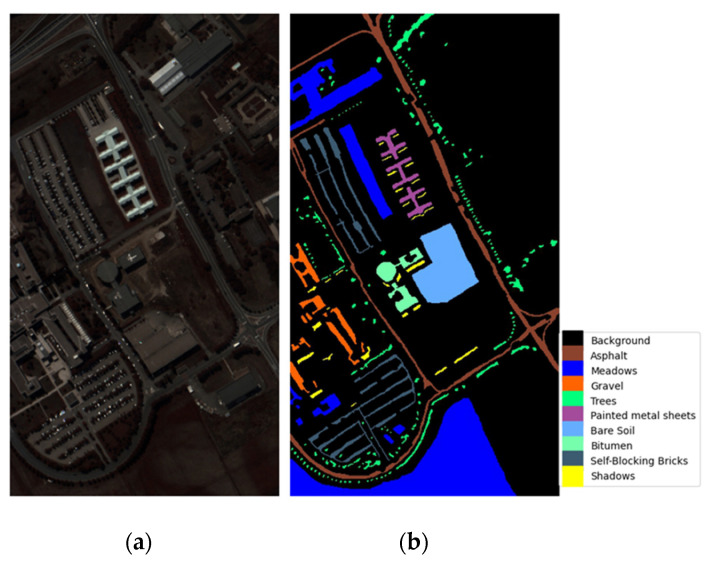
Visualization of the Pavia University (PU) dataset. (**a**) Pseudo-color image for the dataset. (**b**) Ground-truth map for the dataset.

*Pavia University dataset:* This dataset was collected in 2001 at Pavia University in northern Italy using the reflection optical system imaging spectrometer (ROSIS) sensor. It consists of 115 bands with wavelengths ranging from 0.43 to 0.86 microns. The image includes 610 × 340 pixels and nine land cover classes with a spatial resolution of 1.3 m. In the experiment, 12 noisy bands were discarded, and the remaining 103 bands were used for testing. Pseudo-color images and ground-truth classification maps are shown in Figure 6a,b.

**Figure 7:**
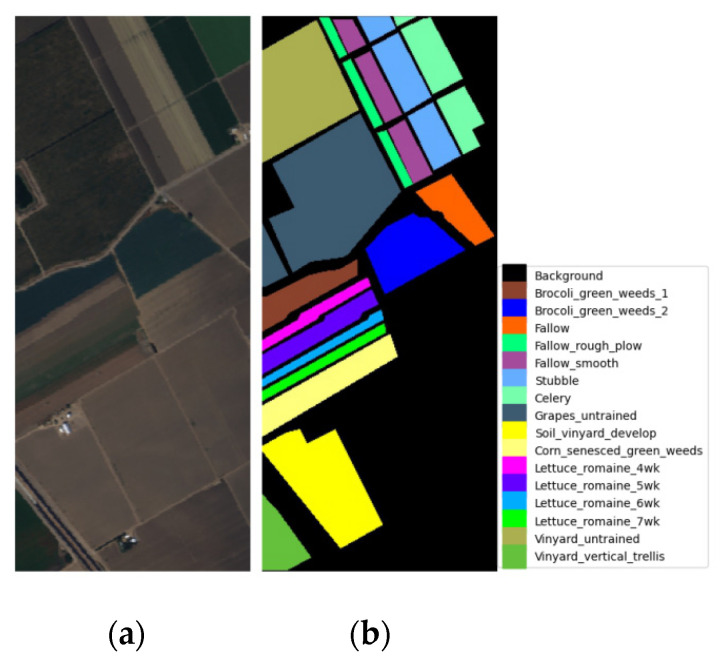
Visualization of the Salinas (SA) dataset. (**a**) Pseudo-color image for the dataset. (**b**) Ground-truth map for the dataset.

*Salinas dataset*: This dataset was collected by the 224-band AVIRIS sensor in the Salinas Valley of California. After discarding the noise bands [108–112, 154–167], and 224, there are 204 spectral bands left. The image consists of 512 × 217 pixels and 16 land cover classes. The pseudo-color image and the ground-truth map are shown in Figure 7a and b, respectively.

### 3.2. Evaluation Indicators

Three evaluation indicators, i.e., overall accuracy (OA), average accuracy (AA), and kappa coefficient (κ), are used in the experiments for HSI classification. OA, AA, and κ can be formulated as in Equation (11):

(11) $$OA=\frac{TP+TN}{TP+TN+FP+FN}\;AA=\frac{1}{C}\sum_{i=1}^{C}\frac{TP_{i}}{TP_{i}+FP_{i}}\;\kappa =\frac{p_{o}-p_{e}}{1-p_{e}}$$

where TP is the true positive, TN is the true negative, FP is the false positive, FN is the false negative, *C* is the number of classes, and *TP_i_* and *FP_i_* are the true positive and the false positive of class *i*, respectively. In addition, $p_{o}$ is the observed precision and $p_{e}$ is the random precision. In classification problems, $p_{o}$ can be calculated by OA, while $p_{e}$ can be calculated by the marginal probability of the class.

### 3.3. Parameter Analysis

For HSI classification, the spatial size of the input data (window) and the batch size used in the training process have a significant impact on the classification performance. This section analyzes them.

**Figure 8:**
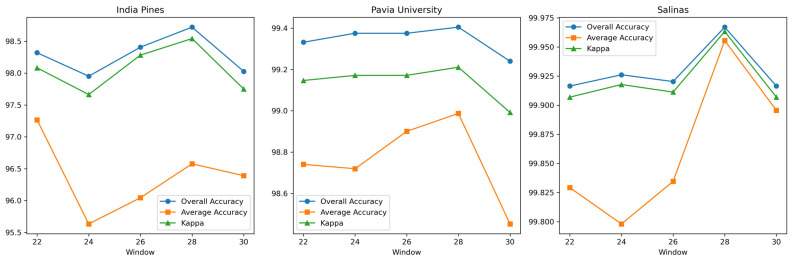
The impact of different input sizes on OA, AA, and κ obtained by the proposed DBMamba.

**Figure 9:**
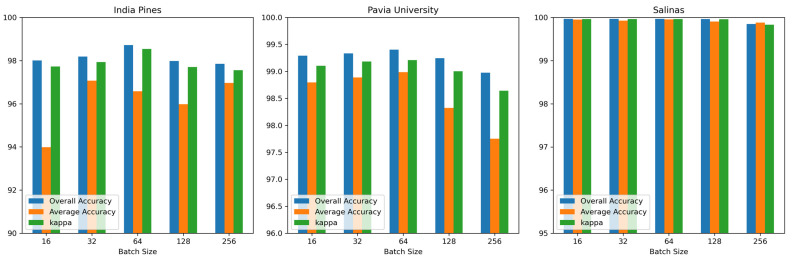
The impact of different batch sizes on OA, AA, and κ obtained by the proposed DBMamba for the three datasets.

**Figure 10:**
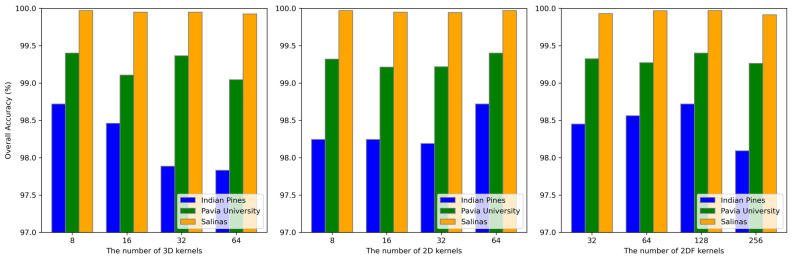
The impact of different number of kernels on OA, AA, and κ obtained by the proposed DBMamba for the three datasets.

**Figure 11:**
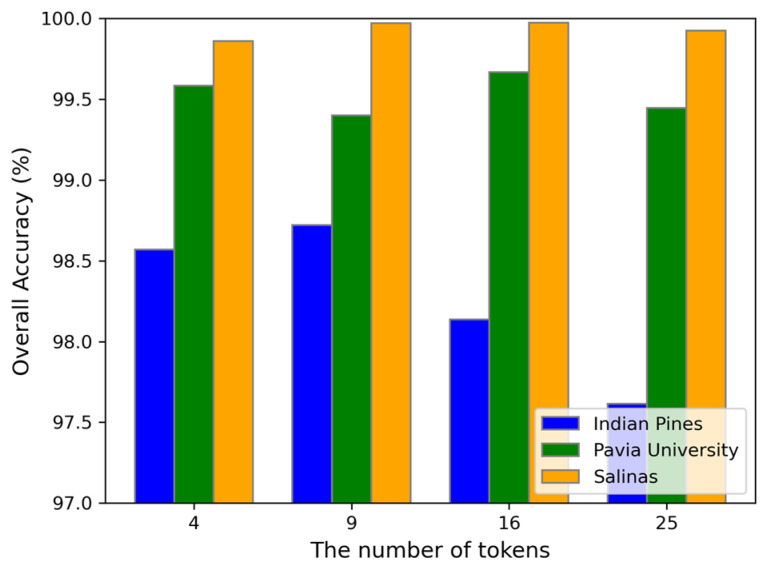
The impact of different number of tokens on OA obtained by the proposed DBMamba for the three datasets.

(1) Window: To explore the optimal input space size of the three datasets for our proposed network, DBMamba, the input space size is set to {20, 22, 24, 26, 28, 30} in sequence for experiments, while other parameters are set to fixed values. The experimental results are shown in Figure 8. As can be seen in Figure 8, although the OA value for the Indian Pines and Salinas datasets fluctuates, it shows an overall trend of first increasing and then decreasing as the input space size increases, with a maximum value of 28. For the Pavia University dataset, as the size of the input space increases, the value of OA first increases steadily and then decreases, also reaching the highest value at 28. At the same time, when the input size is 28, this method has the highest values of OA, AA, and κ on the three datasets. From the above, this paper adopts 28 × 28 as the input size of the proposed DBMamba model.

(2) Batch size: Figure 9 shows the effect of batch size on OA, AA, and κ obtained by the proposed DBMamba for the three datasets. The batch size is selected from {16, 32, 64, 128, 256}. Obviously, when the batch size is set to 64, the optimal OA is obtained.

(3) The impact of different numbers of 3D and 2D convolutional kernels on the OA is shown in Figure 10. The 3D kernels and 2D kernels are used to extract spectral–spatial features and spatial features, respectively, while the 2DF kernels are used for feature fusion. For the Indian Pines dataset, the classification accuracy decreases as the number of 3D kernels increases, but increases with the number of 2D kernels, reaching the highest value with 8 3D kernels and 64 2D kernels. For the Pavia University and Salinas datasets, the highest accuracy is also achieved with 8 3D kernels and 64 2D kernels. Furthermore, as the number of 2DF kernels for feature fusion increases, the OA values for all the three datasets improve, with the maximum value reached at 128 kernels. This demonstrates the effectiveness of feature fusion.

(4) The number of tokens also affects the classification performance of the model. We set the optimal input space to 28 and varied the number of tokens as {4, 9, 16, 25} to conduct experiments on the three datasets. Figure 11 illustrates the impact of the number of tokens on the OA. For the Indian Pines dataset, as the number of tokens increases, the OA first increases and then decreases, reaching its maximum when the number of the tokens is 9. For the Pavia University dataset, while the OA is not the highest at 9 tokens, the difference from the highest value is minimal. For the Salinas dataset, the OA values at 9 and 16 tokens are very close. Based on these observations, we chose 9 tokens as the optimal number.

### 3.4. Classification Results and Analysis

In this section, to verify the effectiveness and superiority of the proposed DBMamba, we make comparisons with several traditional methods and deep learning methods, including SVM [21], 1D-CNN [34], 2D-CNN [35], 3D-CNN [38], HybridSN [39], and SSFTT [47]. The comparison methods listed above keep the network parameters and training strategies in the original paper. The number of training and test samples is shown in Table 1. To ensure fair comparisons, samples are randomly selected.

All experiments in this paper are implemented on the Pytorch software platform, using Intel(R) Xeon(R) Platinum 8352V CPU and NVIDIA GeForce RTX 4090 GPU servers. The Adam optimizer is selected, and the learning rate is set to 5 × 10^−4^. For batch training, each mini-batch size is set to 64. Each dataset is trained using 100 training epochs.

#### 3.4.1. Quantitative Results and Analysis

Table 2, Table 3 and Table 4 show the OA, AA, κ, and accuracy of each category of different methods for the Indian Pines, Pavia University, and Salinas datasets.

It can be seen from the tables that, compared with the six other methods, the proposed DBMamba method obtains the highest OA, AA, and κ values. From Table 2, we can clearly see that SVM, 1D-CNN, and 2D-CNN have lower accuracy in the “Alfalfa” and “Oats” classifications because the training samples of the Indian Pines dataset are randomly selected at 10% and the sample size of these categories is relatively small. However, the classification accuracy obtained by DBMamba is relatively uniform, and it has the best classification performance in many categories such as “Alfalfa”, “Corn-notill”, “Corn-mintill”, and “Oats”, which shows the effectiveness and superiority of the DBMamba method. The same situation can be also observed in Table 3 and Table 4.

The results of the experiments show that the proposed bidirectional Mamba outperforms SSFTT. Specifically, multi-head self-attention in the SSFTT plays a key role. It is sensitive to attention, where an outlier or noise in a pixel can influence the overall result. Due to this, the problem of same spectrum–different materials in HSIs could affect the model accuracy. However, our proposed bidirectional Mamba takes into account positional information from both forward and backward directions, which greatly suppresses this issue.

To further verify the effectiveness of the proposed method and its performance under limited training samples, a recent Mamba-based method, HSIMamba [53], and a recent Transformer-based method, CSIL [54], are used for comparison. For each dataset, 50 samples per class were selected as training samples. Table 5 shows the classification results. From the comparison result, it can be seen that the proposed DBMamba outperforms both CSIL and HSIMamba in terms of classification performance. For example, on the Pavia University dataset, the OA, AA, and κ values are all higher than those of the other two methods.

The method proposed in this paper uses PCA for dimensionality reduction of hyperspectral image spectral dimensions. In reference [55], a good idea was proposed to use a 1 × 1 convolution for spectral feature compression, which achieved reasonable results. We replaced the PCA part of the proposed method with a 1 × 1 convolution for dimensionality reduction, keeping the reduction amount and subsequent processing unchanged. Experiments were conducted on three datasets, and the results are shown in Table 6. From Table 6, it can be observed that the 1 × 1 convolution did not perform well in this method, and overfitting occurred during training. This may be due to the fact that using 1 × 1 convolution increased the training parameters, which required more data for training.

#### 3.4.2. Visual Evaluation and Analysis

**Figure 12:**
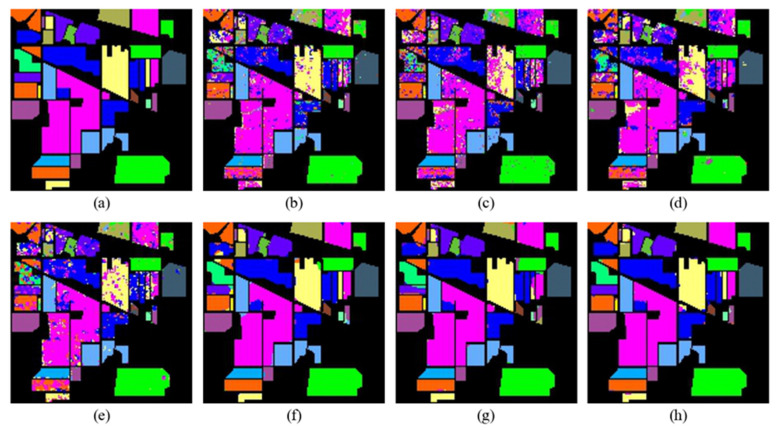
Classification maps of Indian Pines dataset. (**a**) Ground-truth map, (**b**) SVM (OA = 79.90%), (**c**) 1D-CNN (OA = 75.25%), (**d**) 2D-CNN (OA = 77.51%), (**e**) 3D-CNN (OA = 81.49%), (**f**) HybridSN (OA = 96.96%), (**g**) SSFTT (OA = 97.68%), (**h**) Proposed (OA = 98.72%).

**Figure 13:**
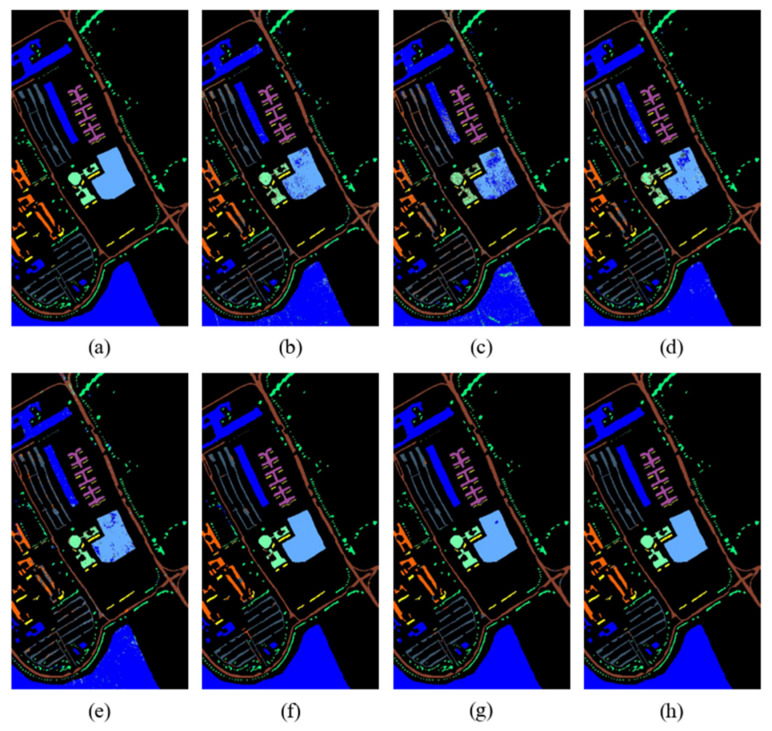
Classification maps of Pavia University dataset. (**a**) Ground-truth map, (**b**) SVM (OA = 93.81%), (**c**) 1D-CNN (OA = 85.82%), (**d**) 2D-CNN (OA = 93.30%), (**e**) 3D-CNN (OA = 93.52%), (**f**) HybridSN (OA = 98.62%), (**g**) SSFTT (OA = 99.25%), (**h**) Proposed (OA = 99.40%).

**Figure 14:**
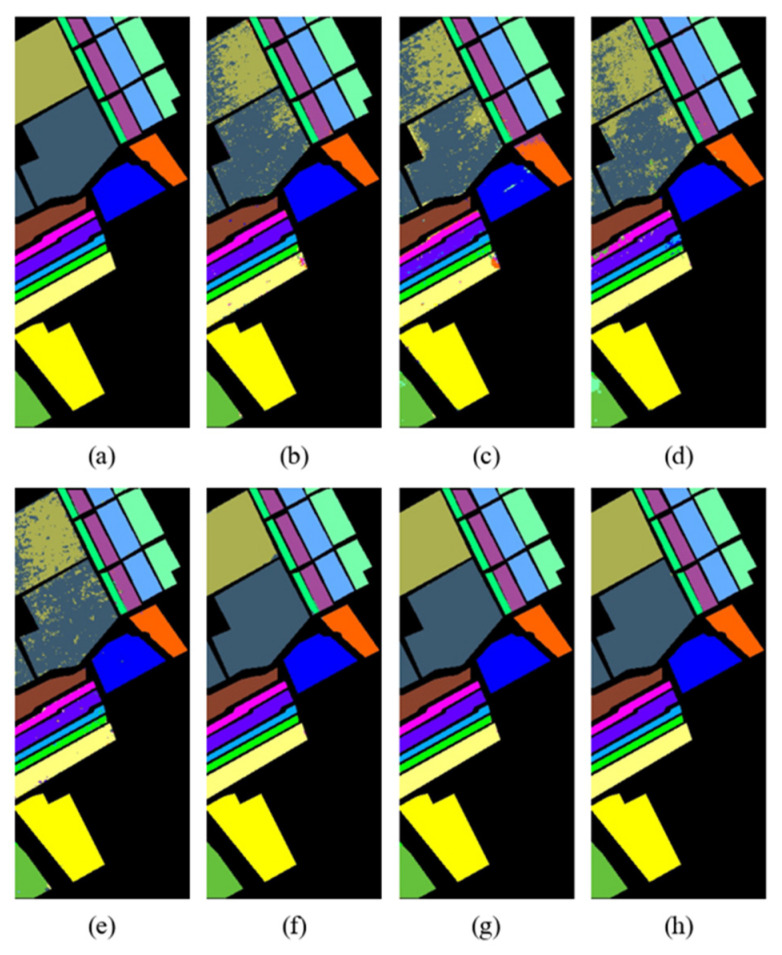
Classification maps of Salinas dataset. (**a**) Ground-truth map, (**b**) SVM (OA = 93.17%), (**c**) 1D-CNN (OA = 90.39%), (**d**) 2D-CNN (OA = 92.63%), (**e**) 3D-CNN (OA = 94.22%), (**f**) HybridSN (OA = 99.82%), (**g**) SSFTT (OA = 99.88%), (**h**) Proposed (OA = 99.97%).

The classification maps of different methods on the Indian Pines, Pavia University, and Salinas datasets are shown in Figure 12, Figure 13 and Figure 14, respectively.

Through visual comparison, it can be seen that the classification maps of the proposed DBMamba are closest to the actual ground-truth maps on all datasets. The classification maps of SVM, 1D-CNN, 2D-CNN, and 3D-CNN are mixed in color and contain a lot of noise, which indirectly shows that these methods cannot achieve accurate classification.

For the Indian Pines dataset, the small purple rectangular block in the center of the left side of each image is a relatively difficult area to distinguish. Almost all the compared methods misclassify it as pink or mixed with green close to its position. The proposed method perfectly identifies the purple area, which also proves the superior performance of the method. For the Pavia University dataset, in the middle light-blue area, several other compared methods show considerable dark-blue clutter. However, the classification map in DBMamba looks very clean and closer to the actual ground-truth map. A similar situation also occurs in the classification map of the Salinas dataset. The excellent classification performance of the proposed method is verified by visual comparison of multiple classification maps.

### 3.5. Complexity Analysis

We compared the proposed DBMamba with 2D-CNN, 3D-CNN, HybridSN, and SSFTT in terms of running time, parameters, and floating-point operations (FLOPs) on the Indian Pines dataset. Ttr and Tte refer to the training time and testing time, respectively. From the results in Table 7, we observe that the proposed DBMamba has fewer parameters than HybridSN. Although our method uses a similar structure to HybridSN, employing 3D-CNN and 2D-CNN to extract hyperspectral features, the introduction of Bim in the proposed DBMamba reduces the number of parameters, proving the effectiveness of Bim. In addition, the proposed DBMamba uses a 3D block with a window size of 28, which is larger than in other methods. However, as shown in reference [52], increasing the input size significantly increases the computational load, slowing down the running time. This explains why the proposed DBMamba takes more time compared to others.

### 3.6. Ablation Analysis

Note that CNNs extract local spectral–spatial features of HSIs through a small receptive field, while the Bim encoder processes sequence data simultaneously from both forward and backward directions to more comprehensively capture the dependencies and features in the data. The proposed DBMamba method combines the advantages of both the CNNs and Bim. To fully demonstrate the effectiveness of the proposed DBMamba, we performed ablation experiments on the Indian Pines dataset according to different components to evaluate the impact on OA, AA, and κ. The experimental results are shown in Table 8.

Specifically, the entire model is split into three components, including a spectral–spatial feature extraction (SSFE) module consisting of a dual-branch structure of 3D and 2D convolutional layers and a module that processes data in the forward and backward directions in Bim. Notably, when Bim is removed, the OA drops by 5.25%. In the second experiment, using a serial arrangement of Conv3D and Conv2D instead of a parallel arrangement resulted in a 0.5% decrease in OA. The third experiment removed the data processing in the backward direction, and the result decreased by 0.67%. Therefore, the ablation experiment fully verifies the effectiveness of the main modules in the proposed DBMamba model.

## 4. Conclusions

This paper proposes a novel method called DBMamba to improve the performance of HSI classification. This method organically combines CNN and Mamba structures to significantly improve the classification accuracy. In the extraction of shallow spectral–spatial features, we adopt a dual-branch structure. The first branch mainly uses 3D-CNN to extract spectral–spatial features from HSIs, and the second branch uses 2D-CNN to extract spatial features. The features obtained from the two branches are fused and converted into a token sequence, and position encoding is added to it. In order to make full use of the contextual information of feature sequences and enhance the ability of Mamba to perceive the position of image features, we employ bidirectional sequence modeling to process the feature sequences from both forward and backward directions, thereby improving the overall performance of the model. Comparative experiments with existing classification methods are performed on three public hyperspectral datasets. The experimental results confirm the effectiveness and superiority of the DBMamba method. At the same time, although the proposed DBMamba method achieves excellent classification performance, there is room for improvement in terms of model complexity, for example, by reducing the number of parameters to shorten runtime.

Future research will focus on designing an end-to-end lightweight Mamba architecture to reduce the computational resource consumption and combining the idea of cross-scanning with Mamba to improve HSI classification accuracy. In addition, Mamba has the advantage of fast response in processing long sequence tasks, which provides new ideas for designing classification models for the joint fusion of hyperspectral and lidar data.

## Figures and Tables

**Table 1:** Numbers of training and test samples in the India Pines dataset, Pavia University dataset, and Pavia University dataset.

No.	Indian Pines			Pavia University			Salinas		
	Class	Training	Test	Class	Training	Test	Class	Training	Test
1	Alfalfa	5	41	Asphalt	332	6299	Green_weeds_1	100	1909
2	Corn-notill	143	1285	Meadows	932	17,717	Green_weeds_2	186	3540
3	Corn-mintill	83	747	Gravel	105	1994	Fallow	99	1877
4	Corn	24	213	Trees	153	2911	Rough_plow	70	1324
5	Grass-pasture	48	435	Metal sheets	67	1278	Fallow_smooth	134	2544
6	Grass-tree	73	657	Bare soil	251	4778	Stubble	198	3761
7	Pasture-mowed	3	25	Bitumen	67	1263	Celery	179	3400
8	Hay-windrowed	48	430	Bricks	184	3498	Grapes_untrained	564	10,707
9	Oats	2	18	Shadows	47	900	Vinyard_develop	310	5893
10	Soybean-notill	97	875	——	——	——	Senesced_green	164	3114
11	Soybean-mintill	245	2210	——	——	——	Romaine_4wk	53	1015
12	Soybean-clean	59	534	——	——	——	Romaine_5wk	96	1831
13	wheat	20	185	——	——	——	Romaine_6wk	46	870
14	woods	126	1139	——	——	——	Romaine_7wk	54	1016
15	Grass-Trees	39	347	——	——	——	Untrained	363	6905
16	Steel-Towers	9	84	——	——	——	Vinyard_vertical	90	1717
-	Total	1024	9225	Total	2138	40,638	Total	2706	51,423

**Table 2:** Classification metrics obtained by different methods for the Indian Pines dataset. The optimal results are bolded.

No.	SVM [21]	1D-CNN [34]	2D-CNN [35]	3D-CNN [38]	HybridSN [39]	SSFTT [47]	Proposed
1	57.89	38.63	85.31	99.56	90.00	**100.00**	**100.00**
2	68.55	73.76	61.58	75.95	97.76	97.68	**98.14**
3	70.29	60.99	78.99	68.41	94.81	96.97	**98.38**
4	56.31	66.66	90.86	69.17	95.88	94.14	**97.67**
5	92.01	74.56	96.98	94.78	**99.18**	94.10	98.61
6	92.70	89.45	97.97	94.46	93.88	99.24	**99.39**
7	74.07	68.18	50.00	78.56	66.67	**92.85**	86.20
8	93.75	89.17	93.66	95.66	99.22	99.53	**99.55**
9	66.67	57.14	65.21	66.66	51.61	**100.00**	**100.00**
10	71.30	65.09	59.15	69.75	96.81	**98.34**	96.50
11	81.74	71.22	73.83	77.91	99.28	98.24	**99.63**
12	78.47	61.08	65.54	72.42	95.44	96.92	**97.39**
13	89.95	90.55	93.87	88.65	98.20	95.85	**99.46**
14	93.24	90.19	94.97	95.61	98.42	98.50	**99.73**
15	68.38	88.02	90.03	85.85	97.16	94.60	**98.85**
16	96.25	97.14	95.18	89.65	77.01	94.04	**100.00**
OA (%)	79.90	75.25	77.51	81.49	96.96	97.68	**98.72**
AA (%)	73.61	68.68	69.88	75.63	97.08	93.73	**97.58**
κ × 100	77.08	71.69	74.14	78.82	96.54	97.35	**98.54**

**Table 3:** Classification metrics obtained by different methods for the Pavia University dataset. The optimal results are bolded.

No.	SVM [21]	1D-CNN [34]	2D-CNN [35]	3D-CNN [38]	HybridSN [39]	SSFTT [47]	Proposed
1	94.48	85.46	92.48	93.32	97.92	99.53	**99.60**
2	95.47	91.59	96.10	96.38	99.53	99.83	**99.89**
3	82.02	72.23	89.50	82.10	92.14	97.09	**97.10**
4	96.97	88.39	99.10	96.56	99.49	98.43	**99.75**
5	97.91	96.88	99.45	**99.92**	99.07	98.45	**99.92**
6	93.91	77.27	93.57	89.48	**100.00**	99.76	**100.00**
7	88.76	77.16	94.66	93.90	98.34	**100.00**	99.29
8	86.65	68.53	77.97	84.11	96.44	95.75	**97.03**
9	**100.00**	99.22	88.83	99.21	99.15	99.19	98.78
OA (%)	93.81	85.82	93.30	93.52	98.62	99.25	**99.40**
AA (%)	91.69	83.25	89.49	91.22	96.59	98.47	**98.99**
κ × 100	91.75	81.17	91.07	91.37	98.17	98.85	**99.21**

**Table 4:** Classification metrics obtained by different methods for the Salinas dataset. The optimal results are bolded.

No.	SVM [21]	1D-CNN [34]	2D-CNN [35]	3D-CNN [38]	HybridSN [39]	SSFTT [47]	Proposed
1	**100.00**	99.94	**100.00**	99.84	**100.00**	**100.00**	**100.00**
2	99.49	99.79	99.60	99.91	99.97	**100.00**	**100.00**
3	98.52	87.68	99.89	99.62	99.76	99.97	**100.00**
4	98.87	97.91	99.25	97.69	98.86	99.26	**99.70**
5	99.28	92.29	98.44	99.00	99.55	99.58	**99.88**
6	99.97	99.65	97.65	99.81	99.83	99.92	**100.00**
7	99.35	96.85	99.82	**99.97**	99.95	99.91	**99.97**
8	81.46	78.79	83.17	85.61	99.43	99.99	**100.00**
9	99.49	97.65	99.62	99.44	**100.00**	99.95	**100.00**
10	96.54	94.82	97.06	97.22	**100.00**	99.84	99.97
11	99.00	98.41	99.69	99.89	99.68	99.71	**100.00**
12	99.83	98.44	99.12	97.72	**100.00**	99.72	99.89
13	98.18	92.90	97.97	95.29	99.96	**100.00**	**100.00**
14	95.65	93.77	96.77	96.85	98.66	98.29	**100.00**
15	82.62	76.12	74.78	83.90	99.82	99.90	**99.91**
16	99.12	99.40	99.30	99.46	**100.00**	**100.00**	**100.00**
OA (%)	93.17	90.39	92.63	94.22	99.82	99.88	**99.97**
AA (%)	96.46	93.72	96.61	96.52	99.82	99.82	**99.96**
κ × 100	92.38	89.29	91.79	93.55	99.81	99.87	**99.96**

**Table 5:** Comparison of experimental results between Transformer-based, Mamba-based methods, and the proposed method.

Datasets	Indian Pines			Pavia University			Salinas		
Methods	DBMamba	CSIL	HSIMamba	DBMamba	CSIL	HSIMamba	DBMamba	CSIL	HSIMamba
OA (%)	96.02	92.65	90.01	97.47	94.32	84.90	99.67	98.49	-
AA (%)	97.11	91.60	89.98	96.69	92.54	88.35	99.59	98.31	-
κ×100	95.43	95.99	89.79	96.63	96.01	80.57	99.64	99.30	-

**Table 6:** The effect of PCA and 1×1 convolution on dimensionality reduction.

Dataset	India Pines		Pavia University		Salinas	
Methods	PCA	1 × 1 Conv	PCA	1 × 1 Conv	PCA	1 × 1 Conv
OA (%)	98.72	88.46	99.40	74.66	99.97	96.62
AA (%)	97.58	85.48	98.99	62.39	99.96	94.70
κ × 100	98.54	86.85	99.21	65.88	99.96	96.24

**Table 7:** Comparison of parameters, FLOPs, and running time on the Indian Pines dataset.

Metrics	2D-CNN	3D-CNN	HybridSN	SSFTT	DBMamba
Parameters (M)	0.295	0.32	4.846	0.148	4.338
FLOPs (M)	0.489	0.52	50.822	11.403	55.362
Ttr (s)	135	170	359.8	137	369
Tte (s)	3.23	4.90	7.92	5.19	7.95

**Table 8:** Ablation studies on different components for the Indian Pines dataset (accuracy in %). The optimal results are bolded.

Cases	Components			Indicators		
	SSFE	Forward	Backward	OA (%)	AA (%)	κ × 100
1	√	×	×	93.47	90.89	92.53
2	Conv3D + Conv2D	√	√	98.22	96.17	97.97
3	√	√	×	98.05	94.95	97.78
4	√	√	√	**98.72**	**97.58**	**98.54**

## Data Availability

Publicly available datasets were analyzed in this study. These data can be found here: https://www.ehu.eus/ccwintco/index.php/Hyperspectral_Remote_Sensing_Scenes (accessed on 23 September 2024).
